# AI-guided DVT diagnosis in primary care: protocol for cohort with qualitative assessment

**DOI:** 10.3399/BJGPO.2024.0165

**Published:** 2024-10-30

**Authors:** Kerstin Nothnagel, Alastair Hay, Jessica Watson, Jonathan Banks

**Affiliations:** 1 Bristol Medical School (PHS), Bristol, United Kingdom

**Keywords:** general practice, venous thrombosis, ultrasonography

## Abstract

**Background:**

Deep vein thrombosis (DVT), a formation of blood clots within deep veins, mostly of the proximal lower limb, has an annual incidence of 1–2 per 1000. Patients who are affected by multiple chronic health conditions and who experience limited mobility are at high risk of developing DVT. Traditional DVT diagnosis involves probabilistic assessment in primary care, followed by specialised ultrasound scans (USS), mainly conducted in hospitals. The emergence of point-of-care ultrasound (POCUS), coupled with artificial intelligence (AI) applications, has the potential to expand primary care diagnostic capabilities.

**Aim:**

To assess the accuracy and acceptability of AI-guided POCUS for DVT diagnosis when performed by non-specialists in primary care.

**Design & setting:**

Diagnostic cross-sectional study coupled with a qualitative evaluation conducted at primary care DVT clinics.

**Method:**

First, a diagnostic test accuracy (DTA) study will investigate the accuracy of AI-guided POCUS in 500 individuals with suspected DVT, performed by healthcare assistants (HCAs). The reference standard is the standard of care of USS conducted by sonographers. Second, after receiving both scans, participants will be invited to complete a patient satisfaction survey (PSS). Finally, semi-structured interviews with 20 participants and four HCAs, and three sonographers will explore the acceptability of AI-guided POCUS DVT diagnosis.

**Conclusion:**

This study will rigorously evaluate the accuracy and acceptability of AI-guided POCUS DVT diagnosis conducted by non-specialists in primary care.

## How this fits in

The incidence of DVT is increasing as the global population ages. Current DVT diagnostic pathways require the use of specialist equipment by trained staff. The emergence of AI-guided POCUS could allow any healthcare professional (HCP) to perform DVT scans. If accurate and acceptable, the use of AI-guided POCUS in primary care could reduce demand for secondary care services, increase access to underserved patient groups, and improve NHS efficiency.

## Introduction

The National Institute for Health and Care Excellence (NICE) reports an annual incidence of 1–2 per 1000 for deep vein thrombosis (DVT), with the risk increasing notably after age 60 years.^1^ Additionally, according to Thrombosis UK, venous thromboembolism will affect 1 in 20 individuals over the course of their lifetime.^2^ Historically, diagnostic USS are conducted by specialists in secondary care. NHS innovation initiatives emphasise transitioning the diagnosis and treatment of non-complex DVT patients from secondary to primary care. This shift enhances patient experience by providing treatment closer to home and aims to yield significant cost savings by reducing unnecessary secondary care referrals.^3^

Nationally, performing a DVT diagnostic scan using point-of-care ultrasound (POCUS) in primary care costs approximately £100 per scan. In contrast, the same scan in secondary care costs £150–£300.^3^ In the Bristol, North Somerset, and South Gloucestershire (BNSSG) region, referring patients suspected of DVT to secondary care costs around £300 per referral, while primary care services such as GP Care in Bristol cost approximately £120 per patient.^4^ GP Care’s ultrasound diagnostics for 1868 patients from January–June 2023 saved an estimated £336 240 over 6 months.

High-risk patients, such as those who are housebound or unable to transfer themselves, must still undergo diagnostics in secondary care.^5^ This process burdens patients and their carers with transportation to emergency care, long waiting times, and subsequent referrals, adding transportation expenses and the risk of unnecessary anticoagulation with associated bleeding risks.

The ability to perform DVT diagnostic scans using POCUS in primary care presents a more accessible and cost-effective option. The successful integration of this technology has the potential to enhance DVT diagnostic pathways, offering diagnostics in primary care and community settings, potentially reducing the need for hospital visits and alleviating financial burdens on healthcare systems.^6^ Handheld ultrasound probes supported by artificial intelligence (AI) apps allow healthcare professionals (HCPs) to conduct DVT ultrasound scans (USSs). This AI-guided POCUS could broaden local diagnostic capabilities, extending its reach to underserved patient groups.^7^ The overall project aim is to assess the accuracy and acceptability of AI-guided POCUS DVT diagnosis performed by non-specialists in primary care settings. Specifically, the following objectives will be assessed. Quantitative objectives are to:

- determine the accuracy of AI-guidance ultrasound compared with standard of care sonographer scans.

Qualitative objectives are to:

- explore patient reservations, obstacles, and confidence regarding their experience with AI-guided POCUS for DVT diagnosis in primary care;
- examine HCPs' experiences and confidence in performing the scan.

## Method

This study employs a mixed-method approach, combining quantitative diagnostic test accuracy (DTA) methodology with qualitative interviews, involving patients and primary care staff. The project comprises three key components:

1. A DTA to investigate the accuracy of AI-guided POCUS in individuals with suspected DVT. It involves AI-guided POCUS conducted by healthcare assistants (HCAs), followed by standard USS performed by sonographers at primary care DVT clinics.
2. A patient satisfaction survey (PSS) (Table 1) to assess participants’ experiences of AI-guided POCUS.
3. Qualitative semi-structured interviews to explore the nuances of acceptance and potential resistance towards AI-guided DVT diagnosis.

### Population and recruitment

**Figure 1.**
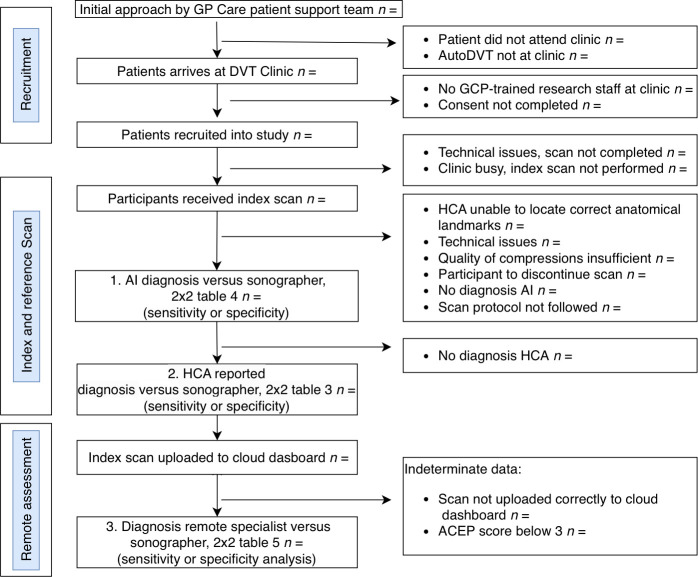
Study flowchart. ACEP = American College of Emergency Physicians. DVT = deep vein thrombosis. GCP = . HCA = healthcare assistant

Patients will be recruited from four primary care DVT clinics operated by GP Care, a social enterprise offering NHS DVT diagnostic services in the BNSSG Integrated Care Board (ICB) region. Eligible patients must qualify for GP Care DVT diagnostic services^4^ and be able to provide informed consent. Study invitations are extended during appointment scheduling and on arrival at the DVT clinic. Patients will be consecutively invited to participate in the study on recruitment days. The flowchart below (Figure 1) outlines the process of recruitment, DTA, and remote diagnosis.

### Consent

On arrival at the DVT clinic, patients will be invited by the research team to participate in the study. If interested, the research team will address questions raised and obtains informed consent (Figure 1).

### Index test

**Figure 2.**
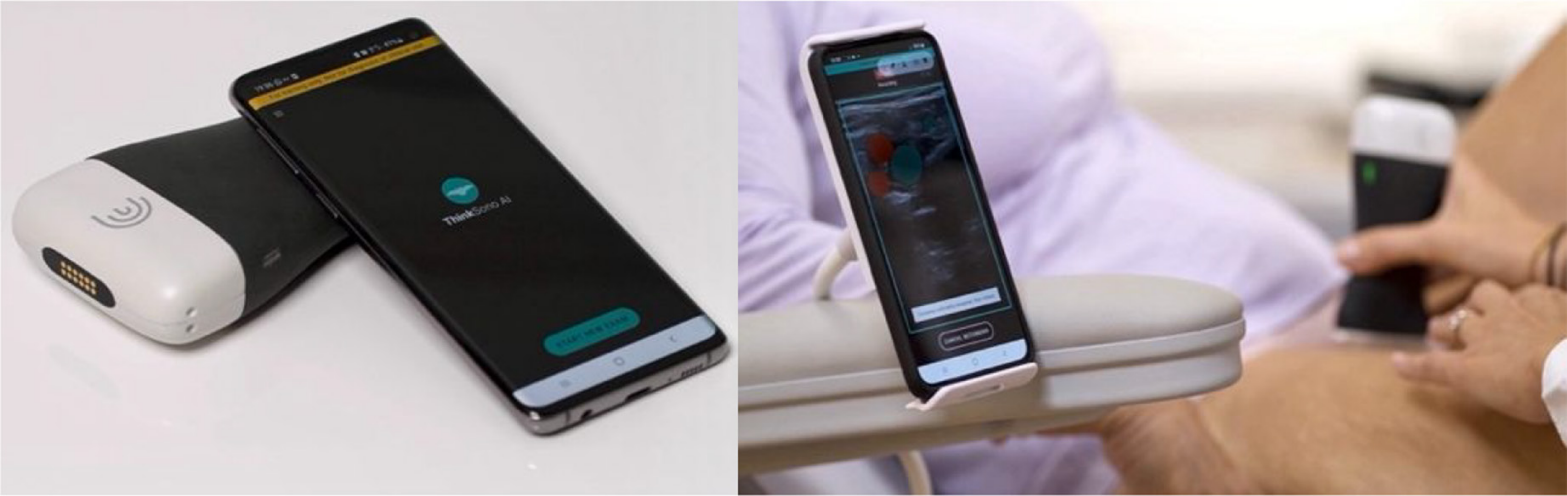
ThinkSono Guidance

Each participant will, first, undergo the index test, which is an AI-guided POCUS performed by an HCA using the ThinkSono Guidance app, as demonstrated in Figure 2 below.^8^ This app, accessible on a smartphone, is linked to a Clarius L7 HD3 linear handheld ultrasound probe.^9^ It provides step-by-step guidance for a three-region POCUS examination of the proximal leg.^10^ When completed, images will be uploaded to a cloud-dashboard for remote review by five independent reviewers (for example, qualified sonographers or radiologists). There will be five independent reviews of each scan.

### Reference test

Participants will receive the standard DVT diagnostic USS recommended by NICE, which involves multiple compression points of the proximal leg.^11^ This scan serves as the reference standard, focusing primarily on the common femoral vein, superficial femoral vein, and popliteal vein, as per NICE guidelines. Data on distal DVTs will be separately reported and recorded on the sonographer’s data collection sheet. Patients with distal DVTs identified during the reference scan, without any additional proximal DVT, will be classified as not having a DVT for the primary analysis, aligning with NICE guidelines that recommend focusing on scanning the proximal leg and provide no treatment recommendations for distal DVTs.

### Blinding

Participants will be informed during the informed consent process that no test results from the index scan will be communicated to them. To preserve blinding of the reference scan sonographer and protect the participant’s privacy, the index scan will be conducted behind medical curtains, and the HCA will be asked to refrain from making verbal comments regarding the procedure or the results.

### Patient satisfaction survey

Each participant is invited to complete a PSS (Table 1) following the completion of both scans. This survey is based on the NHS Friends and Family Test (FFT).^12^

**Table 1.** Patient satisfaction survey

Questions	Response scale					
	Very Good	Good	Neither good nor poor	Poor	Very poor	Don’t know
Thinking about your scan with the ThinkSono Guidance, overall, how was your experience of our service?						
How do you feel about the professionalism of the operator of the ThinkSono Guidance?						
How well were you informed about the purpose of the ThinkSono Guidance scan by the operator?						
Please tell us about anything that we could have done better.						

### POCUS (index test) image review

Each expert assigns ratings using the American College of Emergency Physician (ACEP) image quality scale, which includes the following descriptors: 1 = no recognisable structures; 2 = minimally recognisable structures but insufficient for diagnosis; 3 = minimal criteria met for diagnosis, recognisable structures but with some technical or other flaws; 4 = minimal criteria met for diagnosis, all structures imaged well; and 5 = minimal criteria met for diagnosis, all structures imaged with excellent image quality. An overall score of ≥3 on this scale indicates adequate image quality.^13^ Additionally, each sequence with a score of ≥3 is categorised as having 'compressible' veins (indicating the absence of thrombus), 'incompressible' veins (indicating the possible presence of a thrombus), or requiring a repeat scan ('indeterminate').

### Sample size

We aim to recruit 500 participants to ensure a 95% confidence interval (CI) with a margin of error of ±5% around an assumed sensitivity of 95% (that is, ranging from 90%–100%). This assumption is based on the performance of two-point compression ultrasound in previous studies^14,15^ and an assumed DVT prevalence of 15%, based on GP Care DVT clinic prevalence data.

### Data analysis

#### Study recruitment and participant demographics

Study recruitment will be presented using a flow diagram (Figure 1). Demographics such as age, sex, and postcode within the BNSSG region will be reported in a table.

#### Main analysis

The main analysis will focus on determining the accuracy of remote specialist diagnosis using AI-guided ultrasound images compared with standard of care sonographer scans. Specific objectives include the following:

- evaluating the accuracy of AI-guided DVT diagnosis reported by HCAs compared with standard of care sonographer scans;
- assessing the accuracy of AI-provided DVT diagnosis compared with standard of care sonographer scans;
- identifying the proportion of HCAs' acquired images that are of adequate quality;
- investigating factors associated with inadequate image quality.

#### Assessment of image quality

Inadequate image quality, assessed with an ACEP score <3 according to imaging guidelines,^10^ precludes an overall diagnosis from the remote specialist. We will report the overall proportion of patients for whom images of adequate quality (ACEP score ≥3) could be obtained, relative to initial enrolment.

#### Sensitivity and specificity analysis

Sensitivity and specificity (with 95% CIs) will be calculated for all patients with an ACEP score ≥3 by comparing the remote specialist diagnosis against the sonographer diagnosis.

#### Secondary analyses

Secondary analyses will compare the following:

- HCA-reported results of the AI-guided POCUS with the sonographer diagnosis;
- AI-reported outcome with the sonographer diagnosis.

Results of these comparisons will be presented in Table 2.

**Table 2.** Contingency table for comparison with reference (sonographer) diagnoses

Contingency	Reference (sonographer) positive	Reference (sonographer) negative
Index positive	True positive	False positive
Index negative	False negative	True negative

### Qualitative study

#### Interview process

Semi-structured interviews will be conducted 1–3 weeks after the USS to explore patient, HCA and sonographer perspectives on app-guided POCUS. We aim to achieve information power by interviewing 20 patients and five HCAs who operate the device.^16^ Interviews will be conducted based on patient preferences (face to face, phone, or online) and will cover experiences, challenges, and perspectives on the AI-guided diagnostic pathway.

#### Patient interviews

We aim to interview 20 patients who underwent the AI-guided POCUS to understand their experiences, challenges, and views on the diagnostic process. These interviews will address overall satisfaction, perceived accuracy, and any difficulties encountered.

#### Healthcare assistant interviews

We aim to interview four HCAs to assess their confidence levels and experiences with the AI-guided POCUS. Topics will include technical challenges and confidence in the scan accuracy.

**Sonographer interviews**

We aim to interview three sonographers to explore how AI-guided POCUS may impact their workload, focusing on their role in providing remote diagnoses based on HCA-acquired ultrasound images. Additionally, we will assess their confidence and acceptability towards this shift in responsibility.

### Data analysis

Interviews will be audio-recorded, transcribed, anonymised, and managed in NVivo for organisation, coding, and thematic analysis. Collaboration with patient and public involvement (PPI) advisers will ensure diverse perspectives are considered. The analysis will identify key themes and insights relevant to the study’s objectives.

### Data accessibility

Anonymised transcripts will be available in the University of Bristol (UoB) Data Repository, following participant consent and Data Access Committee evaluation, ensuring transparency and ethical compliance.

### Patient and public involvement

A PPI advisory panel, comprising nine contributors, will actively participate in various aspects of the study, including the design, development of patient information materials, formulation of topic guides, and the crafting of lay summaries.

## Discussion

### Summary

This study will investigate the accuracy and acceptability of AI-guided POCUS DVT diagnosis performed by non-specialists in primary care settings for more patient-centred healthcare practices.

### Strengths and limitations

To our knowledge, this is the largest study of its kind to be conducted in primary care. It uses rigorous methods to ensure independence of index and reference standard scanning in an unselected population attending primary care clinics for investigation of suspected DVT. Further, it combines the use of innovative technology by non-specialist HCPs. If proven accurate, HCAs, who are not registered HCPs, could be widely employed to perform POCUS in primary care settings.

The exclusion of housebound and frail patients and conducting the study in a single geographical area was necessary to constrain resource use but may reduce the generalisability of the findings. Furthermore, it is crucial to acknowledge the dynamic nature of AI advancements in AI capabilities may outpace research findings.

### Implications for research and practice

This study holds promise as a significant advancement in DVT diagnosis. However, it is crucial to critically evaluate its findings, considering both strengths and limitations. The successful integration of this technology has the potential to enhance DVT diagnostic pathways, offering diagnostics in primary care (and in community settings), potentially reducing the need for hospital visits, and alleviating financial burdens on healthcare systems.

The potential benefits of AI-guided POCUS DVT diagnostics include expedited diagnosis, accelerated treatment, enhanced patient outcomes, and cost reduction. AI-guided POCUS is rapid, taking only a few minutes, and can be performed in various community settings, including GP surgeries, patients' homes, or nursing homes.

## Trial registration number

ISRCTN Registry 14795960

## Data

The dataset relied on in this article will be available from the corresponding author in due course on reasonable request.
